# Host Factors and HIV-1 Replication: Clinical Evidence and Potential Therapeutic Approaches

**DOI:** 10.3389/fimmu.2013.00343

**Published:** 2013-10-24

**Authors:** Mariana Santa-Marta, Paula Matos de Brito, Ana Godinho-Santos, Joao Goncalves

**Affiliations:** ^1^URIA-Centro de Patogénese Molecular, Faculdade de Farmácia, Universidade de Lisboa, Lisboa, Portugal; ^2^Instituto de Medicina Molecular, Faculdade de Medicina da Universidade de Lisboa, Lisboa, Portugal

**Keywords:** human immunodeficiency virus, nonprogressors, APOBEC, TRIM, tetherin, SAMHD1, chemokine receptors, chemokine

## Abstract

HIV and human defense mechanisms have co-evolved to counteract each other. In the process of infection, HIV takes advantage of cellular machinery and blocks the action of the host restriction factors (RF). A small subset of HIV+ individuals control HIV infection and progression to AIDS in the absence of treatment. These individuals known as long-term non-progressors (LNTPs) exhibit genetic and immunological characteristics that confer upon them an efficient resistance to infection and/or disease progression. The identification of some of these host factors led to the development of therapeutic approaches that attempted to mimic the natural control of HIV infection. Some of these approaches are currently being tested in clinical trials. While there are many genes which carry mutations and polymorphisms associated with non-progression, this review will be specifically focused on HIV host RF including both the main chemokine receptors and chemokines as well as intracellular RF including, APOBEC, TRIM, tetherin, and SAMHD1. The understanding of molecular profiles and mechanisms present in LTNPs should provide new insights to control HIV infection and contribute to the development of novel therapies against AIDS.

## Introduction

Both pathogens and host have an inherent variability that plays a critical role in the consequences of the infection process. When infected by a specific pathogen, some individuals show no sign of HIV infection or react with moderate manifestations, while others rapidly succumb to the disease. Likewise, heterogeneity in the predisposition to HIV-1 infection has been reported in numerous cohort studies. Approximately 5% of infected patients seem to be unaffected by HIV-1 infection regardless of repeated exposure to the virus by unsafe sexual practices or blood transfusion. Subsequent studies showed the presence of fully replication-competent virus in these long-term non-progressors (LTNPs), which changed the attention to the host.

Understanding what makes non-progressors “immune” to HIV infection is a challenge, as the cohort is not homogeneous. A particular phenotype is the result of specific combinations of factors including the virus strain, each individual immune response and genetic background. Individual variability results from the exchange of genes during meiosis and various mutational events. However, population variability results from natural selection, migration, and bottleneck effects, phenomena that can be analyzed to obtain relevant information about a specific research subject. The study of HIV non-progressors is therefore critical for the understanding of the underlying mechanisms of HIV control that results in low viral replication and/or slow disease progression (>15 years to AIDS) in the absence of therapy (1).

The initial studies on these patients were based on DNA profiling and single nucleotide polymorphism (SNP) genotyping studies that only covered about 0.1% of the entire genome. Although limited in terms of information, they led to the identification of important population-specific polymorphisms that influence HIV-1 infection and progression to disease. One of the most studied genetic variations influencing HIV-1 infection and progression is the Δ32 mutation in the CCR5 gene (2), and SNPs in various chemokine receptors or HLA class I and class II alleles (3, 4). More recently, the use of genome-wide association studies (GWAS) and meta-analysis studies highlighted relevant information on the genetic backgrounds of progressors and non-progressors at a genome-wide level (5).

Non-progressor phenotypes can be explained, at least in part, in terms of host factors that limit HIV infection and disease progression. Here, we review the latest advances in the identification of host factors that determine the vulnerability of cells to viral infection, and discuss their current therapeutic usage and potential. We focus on those factors which restrict the entrance of HIV into the host cell and its later release, such as chemokine co-receptors and their ligands, SAMHD, TRIM, APOBEC, and tetherin.

## Role of Chemokine Receptors and Their Genetic Variability in HIV Infection

**Figure 1:**
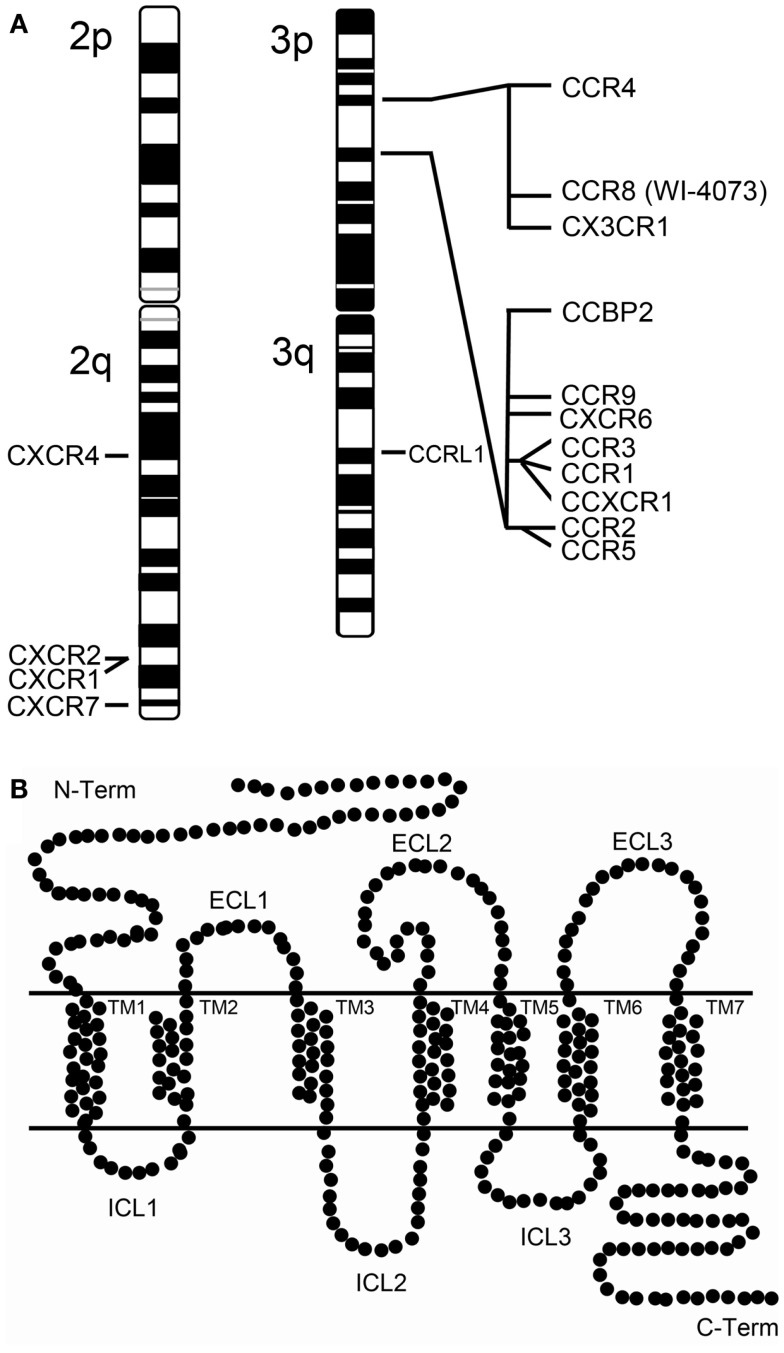
**Human chemokine receptors**. **(A)** Chromosomal map of the human chemokine receptor genes at chromosome 2 and 3. Of notice that the chromosome 3p harbors two chemokine receptor clusters. **(B)** Schematic representation of a chemokine receptor. Chemokine receptors belong to the superfamily of G protein-coupled receptors (GPCRs) which are transmembranar proteins characterized by possessing an extracellular N-terminus and an intracellular C-terminus structure and seven transmembrane α-helices (TM1-TM7) connected extracellularly and intracellularly by six loops (ECL1-ECL3 and ICL1-ICL3).

Several chemokine receptors have been described as mediators of HIV-1 entry. However, CCR5 and CXCR4 are considered the clinically relevant receptors *in vivo* [reviewed in Alkhatib (6)]. CCR5 and CXCR4 are two structurally related chemokine receptors that belong to different classes (C-C and CXC, respectively) of the superfamily of G protein-coupled receptors (GPCRs). GPCRs are transmembrane proteins characterized by seven transmembrane α-helices (TM1-TM7) which are connected by six loops (ECL1-ECL3 and ICL1-ICL3) (Figure 1). CCR5 was first characterized as a receptor for MIP-1α, MIP-1β, and RANTES (7) and later described as a co-receptor for HIV-1 (8). This receptor is highly expressed at the surface of B cells, monocytes, macrophages, dendritic cells (DC), microglial cells, and memory T cells, but rarely in näive CD4+ T cells (9, 10). CXCR4 is also a co-receptor for HIV-1 (11), and its natural ligand is SDF-1/CXCL12. This receptor is expressed on the surface of näive CD4+ T cells, peripheral blood B cells, monocytes, but not on mature macrophages (9, 10). Viruses capable of exploiting CCR5 (R5-tropic) are predominant during the asymptomatic phase of HIV infection, whereas viruses found in late-stage disease use preferentially CXCR4 as their co-receptor (being X4- and R5X4-tropic if they can use both) (12, 13). CXCR4 has an essential role during development (14), which might explain the lack of non-coding variants for CXCR4. The only non-silent CXCR4 polymorphism identified in several HIV-1 infected individuals, the CXCR4 T278C change, was not yet proven to be associated with progression to AIDS (15). However, there are polymorphisms in CCR5 and other chemokine co-receptors that play a key role in natural protection against HIV transmission and progression (16).

The Δ32 mutation in the *CCR5* gene (CCR5Δ32) is probably the most studied genetic variation of a host protein in relation to HIV-1 infection and progression. Due to a 32 bp deletion in the gene sequence, a premature stop codon is introduced, leading to the production of a truncated CCR5 protein. This polymorphism is mostly present in European populations, with higher prevalence in Northern Europe, and is virtually absent in African, Asian, and American Indian populations.

Individuals homozygous for the CCR5Δ32 polymorphism (1% in Europe) do not express CCR5 at the cell-surface and are therefore naturally resistant to the infection by HIV R5-tropic strains, but not by HIV-1 strains that can use a different co-receptor. Indeed, the rare cases of seropositive homozygotic individuals reported so far were infected with CXCR4 HIV strains alone or in combination with a CCR5 tropism (2, 17–25). Heterozygotic individuals are not protected against HIV-1 infection but, in most cohort studies, they have been found to have lower viral loads, slower decrease in the CD4+ T cell count and slower progression to AIDS by an additional 2–3 years when compared to CCR5-wild-type individuals (26–29). The discrepancies found in other studies that failed to correlate CCR5Δ32 heterozygocity with delayed disease progression could be due to small sample size, infection by dual-tropic HIV-1 strains or individual differences at the level of functional expression of CCR5 receptors, which also depends on epigenetics and *trans*-acting factors, for instance (30–32). Despite these contradictory results, meta-analyses of published cohorts associate the CCR5Δ32 allele with lower HIV-1 RNA, decreased risk of progression to AIDS and lower mortality rate in adults (33, 34). GWAS performed on individuals from Euro-CHAVI, MACS cohort, and the International HIV controllers study 2010 further confirmed the protective effect of the CCR5Δ32 allele in viral load control and progression to AIDS (35, 36). Another truncated form of CCR5, the CCR5-m303A, also conferred resistance to HIV-1 infection *in vitro*. CCR5-m303A is a T → A transition at nucleotide 303 which also introduces a premature stop codon resulting in a CCR5 protein that no longer facilitates cell fusion (37). This further supports the putative relevance of CCR5 as a target for HIV therapies.

Several SNPs in the CCR5 *cis*-regulatory region, grouped in at least 10 haplotypes (CCR5-P1 to P10), have been described as changing the course of AIDS. Studying the effects of specific polymorphisms has been challenging due to linkage imbalance across the locus, particularly with CCR2 (Figure 1). Of particular interest is the CCR5-59029 A/G polymorphism, which has been associated with different rates of AIDS progression. HIV-1-infected CCR5-59029 G/G homozygotic individuals progressed slower to AIDS and/or death than HIV-1-infected CCR5-59029 A/A homozygotic individuals (38–40). The frequency of the 59029G allele is significantly increased in exposed seronegative Caucasian men compared to healthy controls (41). G/G, A/G, and A/A promoter genotypes correlated with low, medium, and high viral propagation and CCR5 receptor density, respectively, in *in vitro* studies (42). Promoters containing the 59029G allele showed reduced activity (45%) versus promoters containing the 59029A allele (40). These data strongly suggest that the mechanism underlying the protective effect of the 59029G allele is a lower expression of CCR5. Moreover, the combination of haplotypes CCR5Δ32/CCR5-59029A (in complete linkage disequilibrium) and CCR5wt/CCR5-59029G had a cumulative negative effect on CCR5 expression that conferred an advantage in resisting sexual HIV-1 transmission (41). The CCR5-59353 C allele has been associated with accelerated disease progression and its frequency has been reported to be higher in AIDS patients than in LTNPs (38, 43, 44). On the other hand, Easterbrook et al. (45) observed a correlation between the CCR5-59353 CC genotype and a delay on ∼40% of the subjects in progression to a CD4+ cell count lower than 200; and a higher prevalence of the CCR5-59353C promoter polymorphism among non-progressors compared with those with progressing disease (45). However, they also found that the CCR5-59353 CC genotype was not associated with a delay in the CDC stage IV disease.

The *CCR2* chemokine receptor (also termed CKR2; CCR2A; CCR2B; CD192; MCP-1-R; CC-CKR-2) is an alternative co-receptor for HIV-1 infection that is only used by a few strains. CCR2 constitutively forms homodimers and heterodimers with both CCR5 and CXCR4 (46–50). This gene is located in the chemokine receptor gene cluster region, and it codes for two alternatively spliced transcript variants (CCR2a and CCR2b) (Figure 1). The CCR2 V64I (rs1799864) polymorphism identified with similar frequencies (10–20%) in all ethnicities, has an alteration within the first transmembrane domain of the receptor. This polymorphism does not affect CCR2 co-receptor levels of expression and activity. It is associated with reduced levels of CXCR4 in PBMCs from healthy donors (51); and with a delayed progression to AIDS or death (52–56). However, it does not confer any protection against HIV-1 transmission (57), as exposed uninfected individuals present the same CCR2-64I genotype frequency as HIV-infected individuals and healthy controls in an Indian cohort (58) and in exposed uninfected individuals from both Thai and Puwmani sex worker cohorts (59). The V64I polymorphism is in linkage disequilibrium with point mutations (59353 T/C; 59402 G/A, and 59653 C/T) located in the CCR5 regulatory region due to their close proximity in the chemokine receptor cluster located in chromosome 3 (Figure 1) (60). These observations suggested that the CCR2 V64I polymorphism might prevent HIV progression due to a side-effect on CCR5 receptor expression. However, no association was found between this SNP and CCR5 downregulation. A post-entry regulatory mechanism such as the one resulting from co-receptor heterodimerization or receptor desensitization, cannot be ruled out. CCR2 V64I polymorphism has been described to interfere with cell-surface location of CCR5 (61) and CXCR4 (51), as well as with the CCR5 to CXCR4 transition (62, 63). It can also modulate the heterodimerization of the co-receptors, thus antagonizing HIV infection during the course of the disease (46, 49, 50, 64). Consistent with this second option, the monoclonal antibody CCR2-01 prevents HIV-1 replication by inducing heterooligomerization of CCR2 with CCR5 or CXCR4 viral co-receptors (64). The protective effect conferred by the V64I polymorphism was confirmed in a meta-analysis of individual patient data of European and African descent (34); and, more recently, by a GWAS where a total of 2554 seroconverters and seroprevalent Caucasians were related to their viral load and disease progression (35). These observations led to the development of alternative anti-HIV-1 therapies that target alternative co-receptors like CCR2 (65).

*CXCR6* (also termed STRL33/BONZO/TYMSTR) is the main co-receptor for the simian immunodeficiency virus (SIV) and a secondary co-receptor for HIV that mediates the fusion of HIV-1 M-tropic and dual-tropic strains to CD4 T+ cells. GWAS of LTNPs of a French Caucasian cohort led to the identification of the CXCR6 rs2234358 polymorphism (66). This was further confirmed in three other cohorts of European descent. This CXCR6 variant alters CXCR6 levels of expression and acts independently from the CCR2–CCR5 loci as individuals carrying the CXCR6 mutation did not present any alteration in their CCR2–CCR5 loci. In addition, its action was not linked to a decrease in viral load, as LTNPs had a similar viral load mean as controls (66). Another CXCR6 variant, the CXCR6-E3K or rs2234355, has been related to an increased survival from Pneumocystis carinii pneumonia (PCP) in African-Americans infected with HIV-1 (67). This polymorphism results in the substitution of an acidic residue by a basic amino acid residue in the third codon of the co-receptor, located extracellularly. This could interfere with the receptor-ligand or receptor-gp120 affinity, or lead to a CXCR6 trafficking problem, decreasing the levels of CXCR6 at the cell-surface (68). However, patients carrying the CXCR6-E3K allele and under highly active anti-retroviral therapy (HAART) show a faster virologic failure (sustained viral load < 200 copies/mL) revealing a harmful effect under HAART (69).

The *CX3CR1* chemokine receptor (also termed CCRL1) is an alternative co-receptor for HIV-1 infection that seems to play an important role in HIV-1-associated dementia, immune cell recruitment, and possibly in infection expansion. The possible role of two non-synonymous SNPs, CX3CR1-V249I (rs3732379), and CX3CR1-T280M (rs3732378), in HIV progression to AIDS remains controversial. Both SNPs were initially associated with a faster disease progression in three HIV-1-infected French cohorts (70): patients with intermediate progression (IMMUNOCO cohort), patients with asymptomatic long-term progression (ALT cohort), and patients with a known date of seroconversion (SEROCO cohort) (71–73). However, in three North American cohorts of HIV-1 seroconverters [D.C. Gay cohort (DCG), the Multicenter AIDS Cohort Study of homosexual men (MACS), and the Multicenter Hemophilia Cohort Study (MHCS)] and in the Genetics of Resistance to Immunodeficiency Virus (GRIV) cohort, which are also representative of Caucasian descent, no association was found between these polymorphisms and disease progression (74, 75). On the other hand, the CX3CR1-V249I was found to be more frequent in Spanish HIV-1-infected LTNPs for more than 15 years in a study with a total of 271 Spaniards (LTNPs, progressors, and uninfected controls) (76) and in another study, patients carrying CX3CR1-V249I or T280M polymorphisms showed an improved immunologic response to HAART (69). The CX3CR1-T280M allele was also associated with higher peripheral CD4+ T cell counts in HIV-infected and healthy subjects, showing that these polymorphisms confer protection in the presence of HAART (54). In summary, the role of CX3CR1 polymorphisms in HIV infection and disease progression remains to be elucidated.

## Cytokines and Chemokine Variants

HIV-1 transmission and progression to AIDS can be influenced by allelic polymorphisms in several chemokine and cytokine genes. Cytokines are small signaling peptides that modulate cell functions by means of matching cell-surface receptors. Chemokines are a group of cytokines especially involved in immunological and inflammatory responses that are ligands to GPCRs and share common structural features.

## CCR5 Ligands

CCR5 ligands can be divided in two groups. MIP-1α (CCL3), MIP-1β (CCL4), and RANTES (CCL5) bind efficiently to CCR5 and are full agonists, while MCP-2, MCP-3, and MCP-4 exhibit diverse efficiency and potency in receptor activation (6). Two additional variants, CCL3L1 and CCL4L1, are encoded by genes arising from the duplication of CCL3 and CCL4, respectively (77).

The genes coding for chemokines MIP-1α (CCL3) and MIP-1β (CCL4) are clustered together within a 47-kb region on chromosome 17q12 (77). These are potent chemokines produced by a variety of cell types, such as macrophages, NK cells, fibroblasts, and T cells that stand as natural ligands for the primary HIV-1 co-receptor CCR5 (8, 78). These ligands decrease HIV-1 R5-trophic infection by desensitizing the CCR5 receptor (79). Saha and collaborators have shown that CD4+ T cells from 6 LTNPs produce high levels of MIP-1α and MIP-1β in comparison with AIDS subjects, who produce extremely low amounts of these chemokines (80).

One of the isoforms of MIP-1α, the CCL3L1 also known as MIP-1αP, can physically block HIV-1 entry (81). Variations in CCL3L1 copy number are observed among different ethnic groups; people of African descent have more copies when compared with people of European descent (82). High doses of CCL3L1 could affect HIV-1 infection either by (1) inhibiting HIV-1 gp120 binding to CCR5; (2) reducing CCR5 levels at the cell-surface due to receptor internalization; or (3) affecting leukocyte trafficking important for antiviral responses. However, it seems that chemokine dosage is only significant when compared to the average copy number within an ethnic population. A lower CCL3L1 copy number in one individual, compared with the average copy number in their population, is associated with enhanced susceptibility to HIV-1 infection (82, 83). Both CCL3L1 copy number and CCR5-59029 A/G polymorphisms are associated with delayed disease progression among HIV-1 seropositive subjects and repeatedly sexually exposed HIV-1 seronegative individuals from a North Indian population (84).

The RANTES (CCL5) gene is located in chromosome 17, and encodes a chemokine ligand for CCR1, CCR3, and CCR5. This chemokine is able to block the CCR5 co-receptor, inhibit the recycling of internalized CCR5 to the cell-surface, and subsequently suppress HIV-1 infection by R5-strains (8). Thus, some RANTES derivatives, notably N-terminally modified RANTES variants (AOP-, 5P12-, and PSC-RANTES), have been explored as anti-HIV molecules (85–87). PSC-RANTES and 5P12-RANTES have been also explored as a topical microbicide, after their antiviral activity was demonstrated in non-human primate models (88, 89). Furthermore, three SNPs in this gene (−28C to G, −403G to A, and In.1.1C) were reported to play a role in progression to AIDS. The variant alleles 28G and 403A are associated with delayed progression to AIDS by increasing levels of RANTES transcripts in an Asian population (90, 91). Increased RANTES expression may also contribute to reducing rates of CD4+ T-cell depletion, as it was observed among HIV-infected Japanese individuals (90). Another study on the MACS cohort confirmed the protective role of the 403A allele in disease progression but also described it as a risk factor for HIV transmission (91). However, different results were obtained in a study performed in a Spanish cohort (92), which reflects the controversies around chemokine polymorphisms. These discrepancies may be due to the existence of different allelic frequencies across ethnic groups or to a dominant effect of one variant. For instance, the SNP In.1.1C nested within an intronic regulatory sequence shows the opposite effect of previous alleles, as it accelerates the progression to AIDS in African-Americans and European Americans through downregulation of RANTES transcription (93).

## CXCR4 Ligands

The stromal cell-derived factor 1 (SDF-1) (also termed CXCL12) is the only known CXCR4 ligand, and a potent entry inhibitor for X4-tropic HIV-1 strains (94, 95). It down-regulates the levels of CXCR4 co-receptor at the cell-surface (96, 97). An SDF-1 variant (SDF1-3′A) was identified at position 801 in the 3′untranslated region (3′UTR) of the β variant transcript. Conflicting reports exist regarding the role of SDF1-3′A in HIV infection and AIDS. Homozygotes for SDF-3′A progress slower to AIDS in at least three independent studies that analyzed the GRIV cohort, containing 200 non-progressors and 90 fastprogressors; the ALIVE study, containing 2419 HIV-1-infected patients and 435 HIV-1-exposed uninfected individuals; and a cohort of 12 LTNPs and 12 rapid progressors recruited at the Immunodeficiency Services Clinic at the Erie County Medical Center (98–100). However, other studies found no correlation between the SDF1-3′A allele and disease progression (101–104). Once again the different results obtained in the different studies are probably related to the type of sample, the different readouts and the fact that the *SDF1-3*′*A/3*′*A* effect is recessive, and therefore probably underrepresented in some studies.

## Chemokine Receptor-Based Therapy

Therapeutic targeting at an early phase (pre-integration) of the HIV-1 life cycle is expected to be more effective than acting at later stages of viral replication (post-integration). Early-stage intervention could reduce: (1) the integration of HIV into the host’s DNA as a provirus, and the subsequent establishment of cellular reservoirs of latent virus; and (2) the emergence of viral resistance due to viral mutations.

The observation that CCR5Δ32 delays or prevents HIV-1 infection without affecting health encouraged the development of related anti-AIDS therapeutic strategies, from the disruption of the virus-CCR5 interaction to the inhibition of expression of functional CCR5 co-receptors (summarized in Tables 1 and 2). Both competitive and allosteric entry inhibitors have been designed to disrupt the binding of the virus to CCR5. Competitive inhibitors developed include derivatives of natural ligands of CCR5 (RANTES) and anti-CCR5 monoclonal antibodies. Beyond their ability to compete with the viral Env protein for CCR5 binding, chemokine derivatives can also exert their antiviral activity by inducing internalization of CCR5 from the cell-surface (111). However, one drawback of CCR5 ligand derivatives is the undesired agonistic effect on CCR5. Allosteric inhibitors are small molecules that do not compete with the virus to bind CCR5. Instead, upon binding to a hydrophobic pocket in the transmembrane domain of CCR5, they induce a conformational change of the extracellular loops required for HIV entry. This different approach to inhibit HIV-1 entry has been shown to be very successful giving rise to several compounds that efficiently inhibit HIV-1 replication *in vitro* and *in vivo* (Table 1), including maraviroc and enfuvirtide, the two entry inhibitors approved by the FDA for HIV-1-infected patients (112). Monoclonal antibodies, engineered to block HIV-1 infection without affecting CCR5-mediated signaling, can be administered less frequently than chemokine derivatives and small-molecule inhibitors. The PRO140 monoclonal antibody, for example, has been demonstrated to significantly reduce viral load of patients (110, 113).

**Table 1:** **CCR5-directed therapies**.

Entry inhibitors		
Inhibitor	Development phase	Reference
**ALLOSTERIC INHIBITORS**		
**Aplaviroc** (GW873140)	Terminated at phase 2b (idiosyncratic hepatotoxicity)	Nichols et al. (105)
**Vicriviroc** (SCH-417690, SCH-D)	Stopped at phase 3 (failure to demonstrate superiority to optimized background therapy)	Caseiro et al. (106)
Cenicriviroc* (TBR-652)	Phase 2	Klibanov et al. (107), Lalezari et al. (108), and Marier et al. (109)
**Maraviroc** (UK-427857)	FDA approved	
**COMPETITIVE INHIBITORS**		
AOP-RANTES	Pre-clinical	Toossi et al. (87)
PSC-RANTES	Pre-clinical	Hartley et al. (86)
5P12-RANTES	Pre-clinical	Gaertner et al. (85)
PRO140	Phase 2	Jacobson et al. (110)

*Dual CCR5/CCR2 antagonist.

**Table 2:** **CCR5 gene therapy strategies**.

CCR5 gene therapy		
GT mechanism	Development phase	Reference
siRNA-mediated knockdown	Pre-clinical	Kim et al. (115)
shRNA-mediated knockdown	Pre-clinical	Shimizu et al. (120)
RBZ- mediated knockdown	Pre-clinical	DiGiusto et al. (114)
ZFN-gene-editing	Phase 1/2	Clinicaltrials.gov
		NCT00842634
		NCT01252641
		NCT01044654

In addition, several gene therapy (GT) approaches have been developed to inhibit CCR5 expression. CCR5 expression has been successfully repressed in different models at a gene-editing level by means of zinc-finger nucleases (ZFN); at the RNA level by means of RNA interference or ribozymes (RZB); and at the protein level by means of intrabodies (114–117) (Table 2). The possible efficacy of CCR5-targeting GT to cure AIDS has been strongly supported by the results of the “Berlin patient” who still has no detectable HIV-1 after receiving a hematopoietic stem/progenitor cell (HSC) transplantation from a CCR5Δ32 HLA-matched donor 6 years ago despite discontinuing antiviral therapy (118, 119). Of note, the risks associated to allogenic (i.e., from donors) transplantation, which implicates chemotherapy and radiation, and the low number of CCR5Δ32 homozygotic HLA-matched donors limits the widespread application of this approach. Alternative strategies are to create autologous (i.e., self-donation) CCR5^−/−^ stem cells or CD4+ T cells to be engrafted to the patients. RNAi-based therapies can be achieved either by delivery of siRNA, with a transient effect, or by shRNA lentiviral vectors with stable effects. Both strategies have been shown to be valid. Specific delivery of siRNA against CCR5 to T cells and macrophages by nanoparticles via an antibody to the LFA-1 integrin reduced HIV-1 loads and CD4+ T cell loss in humanized BLT mice (115). Inhibition of HIV-1 replication was also observed *ex vivo* in differentiated spleenocytes from BLT mice engrafted with human CD34+ HSCs transduced with anti-CCR5 shRNA (120). To maximize the blockage of HIV-1 replication, vectors that combine anti-CCR5 shRNA with other therapeutic targets, like TRIM5α (discussed below), have been tested. A combinatorial lentiviral vector with a CCR5 ribozyme, Tat/Rev shRNA, and a TAR decoy was tested in AIDS lymphoma patients (114). The non-toxic expression of vector, shRNA, and ribozyme 24 months following autologous, gene-modified HSC transplantation, established that this therapeutic approach was safe. However, *in vivo* efficacy remains to be proven. Although encouraging results have been reached with these GT approaches, these vectors can be associated with genotoxicity and malignancy. Therefore, the safety of the integrative vector systems needed for stable transgene expression needs to be optimized. These vectors can integrate in undesired places of the host’s genome, inactivating essential genes or activating deleterious genes, such as proto-oncogenes. Additionally, sufficient levels of anti-CCR5 activity need to be reached and maintained. Gene-editing with ZFNs has been tested to overcome these problems. ZFNs are engineered proteins composed by a DNA-binding zinc-finger protein fused to the catalytic domain of a *Fok*I restriction endonuclease (121). Upon binding to the targeted DNA sequence, the ZFN introduces DNA double-strand breaks that are repaired by the error-prone NHEJ repair pathway of the host cell. This usually introduces permanent nucleotide insertions and deletions in a gene sequence that will produce a non-functional protein. Thus, ZFNs only need to be transiently expressed to achieve a permanent modification of the CCR5 gene, and this can be achieved by means of standard DNA delivery (nucleofection) or non-integrating vectors systems. Perez and collaborators reported that a CCR5-targeted ZFN disrupted approximately 50% of CCR5 alleles in primary human CD4+ T cells (116). By using new immunosuppressed mouse models that widely accept heterologous cells, such as the NOG mouse model [NOD/SCID/IL2rgamma (null)], these CCR5-modified T cells were able to grow stably and block R5-tropic HIV-1 replication both *in vitro* and *in vivo*. Disruption of CCR5 by means of ZFNs has also been achieved *ex vivo* in human CD34+ HSCs (122). CCR5-modified HSCs retained their ability to engraft NOG mice and displayed normal multi-lineage differentiation. Mice engrafted with CCR5-modified HSC had significantly lower HIV-1 levels and higher CD4+ T cells counts than control mice. The safety and tolerability of CCR5- modified CD4+ T cells are being tested in one completed and two ongoing clinical trials (Table 2).

## Restriction Factors

**Figure 2:**
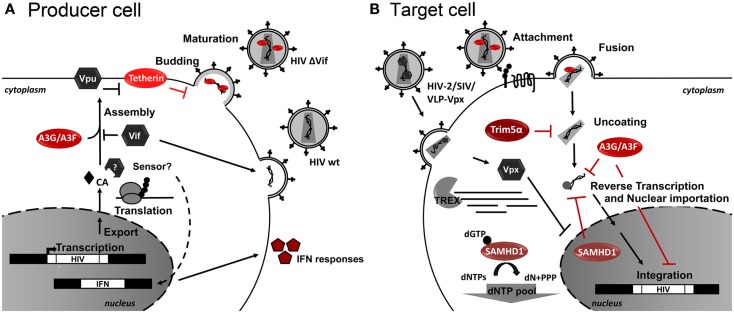
**Host restriction factors and their action during HIV-1 replication**. Schematic representation of **(A)** HIV-1-infected producer cell, and **(B)** HIV-1 target cell. Cellular restriction factors are represented by red ovals, and viral counterpartners are represented by gray hexagons. Black arrows represent the course of viral replication and actions. Broken arrows represent inhibition. Question marks (?) represent unresolved questions.

Restriction Factors (RFs) are cellular proteins that can restrict or block viral replication in a cell-specific way. Generally RFs are not sufficient to block HIV-1 replication as HIV-1 developed several countermeasures to abolish its activity via virus-specific proteins and degradation by the proteasome. RFs are part of the innate immune response and normally respond to Interferon (IFN) stimulation. Several RFs of HIV replication have been identified, which act at several key steps of the HIV-1 life cycle. As RFs naturally control HIV infection, it is conceivable that RF genetic alterations or levels of expression are related to differences in HIV progression. Consequently, RF-based therapeutic strategies can be envisioned to control HIV replication. For an overview of the retroviral life cycle and the RF discussed herein see Figure 2.

## APOBEC Protein Family

Apolipoprotein B-editing catalytic polypeptide 3 proteins (APOBEC3, A3) are members of the cytidine deaminase family that share a common structure. During evolution, several members of the human APOBEC3 gene cluster (A3B, A3DE, A3F, and A3G) suffered duplication and/or recombination [reviewed in Conticello (123)]. With the exception of A3C, all members of the A3 family have anti-retroviral activity.

APOBEC protein expression and activity must be strictly regulated in order to maintain genome stability and cellular metabolism, as overexpression leads to the appearance of cancer (124). A3G and A3F are highly potent single-stranded DNA (ssDNA) cytidine deaminases. These editing enzymes likely evolved to control the replication of endogenous retroelements and exogenous retrovirus, including HIV-1. A3G specifically restricts the replication of incoming viruses in resting CD4 T cells and monocyte-derived macrophages (MDM cells) (125–127). A3G and A3F activity is mainly regulated through their association with other cellular factors, and switches from an active low-molecular-mass (LMM) ribonucleoprotein complex to an inactive high-molecular-mass (HMM) complex (128). Their activity can also be modulated by other complementary mechanisms, including tissue- and stage-specific signaling, transcriptional regulation, subcellular localization, posttranslational modifications, interaction with specific cofactors, and accessibility of the target sequence [reviewed in Smith et al. (129)].

Both A3G and A3F RF need to be incorporated into the viral particle to be capable of exerting their restriction phenotype in the target cell (130, 131). However, recent studies indicate that A3G does not always need to be packaged in the viral particle to exert its antiviral function, as endogenous LMM A3G can restrict HIV in resting CD4 T cells (132, 133). A3G antiviral activity can either be deaminase-dependent or deaminase-independent. For deaminase-dependent activity, A3G exerts its action in target cells during reverse transcription of the growing minus strand of viral DNA, which is deaminated independently of the reverse transcriptase (134, 135). The resulting dU-rich transcripts have two possible fates. They are either degraded by the cellular uracyl-DNA-glycosylase (UDG), causing the failure of reverse transcription (136, 137), or yield G-to-A hypermutated proviruses that are largely non-functional, with the consequent reduction in viral fitness (125, 134, 138–141). More recently, the deaminase-independent restriction activity of A3G against HIV-1 was discovered as cells bearing catalytically inactive A3G mutants kept their ability to block HIV-1 infection (142, 143). In addition, A3G inhibits several steps of viral cDNA synthesis and integration by: (1) reducing the efficiency of plus-strand transfer (144); (2) reducing tRNALys3-priming and initiation of viral DNA synthesis (145–147); and (3) interfering with reverse transcription, DNA elongation, and proviral integration (146, 148–150). A3F showed similar, but more pronounced, effects on HIV-1 infection (149–152).

HIV escapes the cellular restriction exerted by A3G and A3F by expressing the viral infectivity factor (Vif) (153–155). Vif specifically depletes A3G and A3F from the virus-producing cells by inducing its proteasomal degradation (137, 156–161). Vif is part of the RING-finger E3-ubiquitin complex with Elongin B (EloB) and C (EloC), Cullin 5 (Cul5), and Ring-box protein 2 (Rbx2) and the recently identified core-binding factor β (CBF-β) (162–165). Vif also reduces A3G translation (131) and competes or directly blocks A3G viral incorporation (166–168), besides blocking A3G catalytic activity (169). A3G overexpression overcomes the action of Vif-positive viruses (159), indicating that higher A3G expression is responsible for the high-incidence of G-A mutations in the proviral DNA from many HIV-1-infected subjects, even in the presence of Vif (157, 170–175). This A3-mediated hypermutation of the proviral genome can confer a selective advantage or disadvantage for viral replication (176) and result in the appearance of either more virulent or innocuous strains (173, 174, 177).

## APOBEC Clinical Studies

Population-based studies have tried to establish a relationship between A3 polymorphisms, expression, and/or activity and the rate of disease progression (174, 175, 178–182). However, the different experimental setups and readouts, sample size, types of populations, host, and viral specific genetics and analysis contribute to data entropy and inconsistent results. An A3G polymorphism identified (H186R or rs8177832) in African-Americans and the 6,892C allele present in European-American populations were associated with accelerated disease progression (174, 178, 181). Nevertheless, protective A3G or A3F polymorphisms have yet to be identified. An extensive study where the genetics of the GRIV cohort were analyzed led to the identification of several new SNPs in APOBEC3G. However, none of them presented any association with AIDS progression in this cohort (183). In other studies, A3G/A3F mRNA levels were measured as a readout of A3G/A3F expression in activated cells (179) or in non-activated cells (181, 184), and they were compared with markers of disease progression such as viral load and CD4+ T cell counts (179–181, 185, 186). They found an inverse correlation between A3G mRNA levels and disease progression in LTNPs (179), but not in progressors (184). Other groups measured proviral genome hypermutation as a readout for A3G/A3F activity (174, 187–189). Some studies found a correlation between hypermutation and high CD4+ T cell counts (188) or reduced plasma RNA levels (174), while another report did not find any relationship between hypermutation and either viral load or CD4+ T cell counts (189). In addition, a study comparing elite suppressors and patients under antiviral treatment showed no statistical differences between their hypermutation frequencies (187). As deamination is not the sole A3 antiviral mechanism, deamination (catalytic activity) might not represent A3G/A3F antiviral activity properly and might explain the different conclusions obtained from different studies. For instance, studies on elite suppressors showed that these subjects have lower levels of integrated proviral DNA and generated more proviral 2-LTR forms than HIV-1 patients on treatment, probably due to cell-specific, integrase-independent mechanisms (190, 191). More recently, a study with 19 anti-retroviral-naïve HIV-positive patients [12 LTNP (<5000 RNA copies/ml over the prior 5 years) and seven non-controllers (>10000 copies/ml)] established a relationship between the control of HIV-1 infection by elite suppressors and A3G and A3F expression and activity (192).

These observations support the possible use of pharmacological modifiers of A3 expression as an alternative strategy to increase the natural protection against HIV replication. The upregulation of A3G expression can be mediated by the stimulation of CCR5 and CD40 (part of a major co-stimulatory pathway) with CCL3 and CD40L (CD154) chemokines, respectively, as well as by the heat shock protein 70 (HSP70). In fact, the use of HSP70 as a preventive measure was already tested in rhesus macaques with success (193). CCR6 ligands also increase the natural protection of CCR6+ cells against HIV by inducing A3G expression (194). Since the A3G activity is tightly regulated in the HMM complexes, an increase in A3G expression might not be enough to overcome HIV replication in all cells. Thus, the use of HMM inhibitors might serve as a complement to this strategy, as they would activate A3G (125, 127, 133). However, these strategies must be strictly regulated to avoid unwanted side-effects.

The Vif-A3G, Vif-Cullin5, or Vif-CBF-β interaction sites are also promising sites for the development of new anti-HIV molecules such as the RN-18 (195, 196) or the 4BL intrabody (197), that specifically target the HIV-1 Vif protein. However, drugs that specifically interfere with A3G degradation by targeting Vif cellular partners are preferable to avoid the generation of resistant mutants, e.g., the IMB-26/35 small molecules that bind A3G and block its Vif-mediated degradation (198). Alternative strategies to inhibit HIV by enhancing viral incorporation of A3G into HIV-1 Vif+ viral particles have been tested: theVpr14-88-Apobec3G fusion protein strategy (199) and the Nef7-A3G fusion protein strategy (200). More recently, the use of Chim3, a Vif-dominant negative protein, was shown to block HIV-1 replication by acting at the pre-integration step of HIV-1 (201).

## Tripartite Motif Family Proteins

The TRIM family includes approximately 100 proteins characterized by a highly conserved tripartite motif (TRIM) structure on their amino-terminal region, called RBCC motif. This motif is constituted by a RING (*Really Interesting New Gene*) domain, one or two B-box domains and a coiled-coil domain (CC) (202). The RING domain is a zinc-binding motif with E3-ubiquitin ligase activity which mediates the conjugation of proteins with ubiquitin, small ubiquitin-like modifier (SUMO), or with the ubiquitin-like IFN-stimulated protein of 15 kDa (ISG15) (203). The B-box domains are zinc-finger proteins. The CC is a helical structure important for homo- or heteromeric interactions that lead to the formation of high molecular-mass complexes that could determine the function of TRIM proteins. The diversity of the TRIM family results from the 10 different C-terminal domains that can be found alone or in combination allowing TRIM proteins to be classified in 11 different classes (204). Two of the most common C-terminal domains are the PRY and SPRY domains, which can combine to form a PRYSPRY domain (also known as B30.2). TRIM proteins are involved in several biological processes such as innate immunity, cell differentiation, and transcriptional regulation (203, 205). Several TRIM family members have been identified as HIV-1 RF acting at different steps in the HIV-1 life cycle, namely TRIM5, TRIM11, TRIM15, TRIM19, TRIM22, TRIM31, and TRIM32. However, the TRIM5 proteins are the best studied.

TRIM5 is expressed along the primate lineage and is encoded by the *TRIM5* gene. This gene codes for different TRIM5 isoforms, amongst which only TRIM5α and TRIM5Cyp show antiviral properties (206, 207). TRIM5Cyp from the New World Owl monkey is structurally different from TRIM5α at its C-terminal end, containing a cyclophilin A domain instead of the PRYSPRY domain present in the α isoform. Primate TRIM5α orthologs inhibit several retroviruses and lentiviruses but are ineffective against their own host-specific viruses. For example, while human TRIM5α (huTRIM5) strongly restricts N-tropic murine leukemia virus (N-MLV), it only weakly restricts HIV-1 infection. Rhesus monkey TRIM5α (rhTRIM5) efficiently blocks HIV-1 but not the infection by the autologous simian immunodeficiency virus (SIV_MAC_) (207–209).

It is well-established that TRIM5 proteins block HIV-1 infection at an early-stage of reverse transcription but their exact antiviral mechanism remains unclear. TRIM5 proteins bind to the HIV-1 capsid (CA) and induce its premature disassembly before reverse transcription can occur (207). The biochemical interactions between the CA and TRIM5 proteins are complex and important for their restriction activity. They involve: (1) the binding of their C-terminal domain (B30.2 domain for TRIM5α and cyclophilin A for TRIM5Cyp) to the CA lattice (206, 210–212); and (2) the dimerization and higher-order multimerization of TRIM5 (213–215), which ultimately leads to the formation of an hexameric protein lattice (216). Both coiled-coil and B-box2 domains of TRIM5 are required for its dimerization and multimerization (213, 217–219). In addition to a direct antiviral mechanism, it has been suggested that TRIM5 acts as a pattern recognition receptor that “senses” the CA lattice, leading to the activation of the innate immune response (220). This CA sensing triggers the E3-ubiquitin ligase activity of the RING domain of TRIM5 proteins that, together with the heterodimeric E2 Ubiquitin-conjugating enzyme complex UBC13-UEV1A, generate unattached K63-linked ubiquitin chains, leading to its multimerization and the activation of the TAK1 kinase complex. Subsequently, TAK1 activates NF-κB and AP-1 signaling (220). However, the exact contribution of innate immune response and E3-ubiquitin ligase activity in TRIM5 antiviral activities still needs to be evaluated. Deletion of the RING domain only partially abrogates restriction of HIV-1 by TRIM5 proteins (211, 221) and while proteasome inhibitors prevent TRIM5 blockade on CA disruption and reverse transcription, they do not affect TRIM5 antiviral activity (222, 223).

## Trim5α Genetic-Variants Clinical Studies

Several studies have addressed the relationship between huTRIM5α and its genetic-variants and HIV disease progression to AIDS. However, this is still a controversial subject. The huTRIM5α gene has several SNPs but only two of them have been studied for their effect on disease progression (H43Y huTRIM5α and R136Q huTRIM5α) (224, 225). The huTRIM5α H43Y polymorphism occurs at the RING domain of TRIM5α and may therefore affect its E3-ubiquitin ligase activity (225). *In vitro* assays showed that the 43Y variant exhibits an antiviral activity lower or similar to the 43H variant (224–227). However, the discrepancies among these studies could be due to the different expression systems used, as the H43Y polymorphism shows protective effects against HIV-1 infection in African-Americans and Chinese intravenous drug users (226, 228). However, other epidemiological studies failed to correlate the H43Y polymorphism with resistance to HIV-1 infection or AIDS progression (224, 226, 227, 229). Likewise, and consistent with its lower *in vitro* antiviral activity, a H43Y homozygous genotype is predictive of an accelerated progression to AIDS (230). As the H43Y polymorphism results in different protective effects in different populations, it is conceivable that the genetic background may account for these conflicting results among epidemiological studies. The other huTRIM5α polymorphism (R136Q) occurs at the CC which is, as mentioned, important for TRIM5 protein oligomerization and antiviral activity. The antiviral activity of the 136Q variant is higher than 136R which is consistent with the HIV-1 protective effect observed in both US-based natural history and Pumwani sex workers cohort studies (226, 229). However, this protective effect appears to be dependent on the strain of HIV, as it was only observed after the emergence of X4-strains and not with R5-strains (230). Conversely, Goldschmidt and co-workers were unable to correlate the R136Q polymorphism with disease progression (224). Thus, besides the strong significance of TRIM5 proteins as antiviral factors, more data on genetic polymorphisms needs to be gathered and analyzed in large cohorts. This assumption is crucial to determine the species-specific activity of TRIM5 proteins and how they relate to the innate immunity of different populations.

### TRIM5α-based therapies

TRIM5α is an attractive cellular host protein for HIV-1 gene-based therapies as it acts at a post-entry level, which is a therapeutic advantage as already mentioned. A pioneer study by Anderson and Akkina (231) showed that human macrophages differentiated *in vitro* from CD34^+^ HSCs and transduced with rhesus macaque TRIM5α resisted HIV-1 infection, thus providing the proof of principle that TRIM5α could be used in gene therapy. However, since rhTRIM5 is not human, it would likely elicit an undesirable immune response. On the other hand, the use of human TRIM5α would not trigger these immune responses, but unfortunately it has less potent antiviral activity. To overcome these limitations the hTRIM5α has been engineered. One strategy was to construct a chimeric human-rhesus (HRH) isoform that contains the rhesus macaque TRIM5α 13 aa sequence in place of the human 11 aa region located in the PRYSPRY domain (232). CD34+ HSC transduction with this HRH chimeric isoform originated normal macrophages *in vitro*, normal T cells *in vivo*, and hindered HIV-1 infection of CCR5- and CXCR4-tropic HIV-1 clones. To further increase the efficacy of HIV-1 gene therapy, Anderson and collaborators combined the HRH chimeric isoform with two other transgenes that act at different stages of the HIV-1 life cycle: a CCR5 shRNA (pre-entry) and a *Transactivation* response element TAR decoy (post-integration) (233). This anti-HIV-1 vector displayed complete protection from productive viral infection and integration of multiple HIV-1 strains upon transduction into HIV target cells *in vitro* (233) NOD-RAG1^−/−^IL2rγ^−/−^mice [immunodeficient mice carrying mutations in the recombination activating gene-1 (*Rag1^null^*) and interleukin (IL)-2 receptor common gamma chain (*IL2r*γ*^null^*)] were engrafted, with CD34+ HSCs and transduced with the anti-HIV vector described above. They exhibited normal multi-lineage hematopoiesis and no decrease on human CD4+ T cells levels upon infection with R5 and X4-tropic strains of HIV-1 (234). However, the latter observation was not accompanied by a decrease of plasma viremia, and blockade of HIV-1 infection was only observed in *ex vivo* experiments. Another promising engineered human TRIM5α protein is huTRIM5Cyp, a design inspired by TRIM5Cyp from New World owl monkey, a potent inhibitor of HIV-1 replication (235). This molecule, resulting from the fusion of human TRIM5α and human CypA, blocked CCR5-, and CXCR4-tropic HIV-1 clones and primary isolates of HIV-1 replication in several cell types, including CRFK, Jurkat, and primary T cells (CD4+ T cells and macrophages). HIV-1 infection was also impaired in other humanized immunodeficient mouse line, the NOD-RAG2^−/^-γc^−/−^strain, when these mice were engrafted with CD4+ T cells or CD34+ HSCs transduced with huTRIM5Cyp (235). Interestingly, a higher restriction in HIV-1 infection was achieved by engrafting mice with transduced CD4+ T cells than with transduced CD34+ HSC cells. Additionally, a functional screening of huTRIM5α mutants generated by PCR-based random mutagenesis identified that an R335G mutation efficiently prevented HIV-1 infection *in vitro* (236). Additionally, a functional screening of huTRIM5α mutants generated by PCR-based random mutagenesis identified that an R335G mutation efficiently prevented HIV-1 infection *in vitro* (236). Thus, novel huTRIM5α proteins with few mutations could be engineered to effectively inhibit HIV-1 infection with limited immunogenicity. Mutations could also be achieved *in vivo* by using ZFN as described above for the CCR5 receptor. Overall, these encouraging results confirm the potential of TRIM5α and gene therapy approaches to treat HIV-1 and pave the way for clinical studies, which, to our knowledge, are not underway.

## Tetherin

Tetherin (BST-2/CD317/HM1.24) is a type 2 transmembrane protein anchored by a transmembrane domain near the N-terminus and a glycosylphosphatidylinositol (GPI) anchor at the C-terminal that may be a second transmembrane domain (237, 238). This protein is also composed of an N-cytoplasmic tail and an ectodomain that links the two anchors. Through its GPI anchor, tetherin is located in lipid rafts at the plasma membrane, *trans*-Golgi Network (TGN), and early and recycling endosomes (238–241). The ectodomain contains an α-helical coiled-coil region with cysteine residues that allow the formation of parallel homodimers by disulfide bonds (242–244). The coiled-coil region has structural irregularities believed to provide conformational flexibility (244, 245). Crystal resolution also showed that two tetherin dimers could associate to form a tetramer (243, 244).

Tetherin is constitutively expressed in mature B cells, bone marrow stromal cells, plasma cells, plasmacytoid DC, and some cancer cell lines (242, 246). Its expression can also be induced in several cell lines following stimulation with type-I IFN, IFN-γ, or other pro-inflammatory stimuli (242, 246–249). Tetherin’s cellular expression pattern suggests a role in the development of pre-B cells and tumor invasion. Recently, it was proposed to negatively regulate IFN production by binding to immunoglobulin-like transcript 7 (ITL7) in plasmacytoid DC (250). However, probably the best established physiological function of tetherin is its antiviral activity against various virus families.

Tetherin was first identified as the cellular factor responsible for the inhibition of the spread of *vpu*-defective HIV-1 mature virions, by preventing their release from the cell (251, 252). Tetherin exhibits an antiviral activity against a wide range of enveloped viruses as its main target is the lipid bilayer derived from the host cell (253). Tethered viral particles can remain at the cell-surface or suffer endocytosis and potentially be degraded in a process promoted by Rabring7 (254). It has also been suggested that these tethered virus could influence the cell-to-cell virus transmission which occurs through virological synapses. While two *in vitro* studies reported that tetherin is capable of decreasing cell-to-cell transmission, a third one describes the opposite effect (255–257). A recent *in vivo* study where tetherin reduces viral burden and inhibits pathogenesis supports the hypothesis that tetherin does not favor cell-to-cell transmission (258). However, more studies are needed to clarify the role of tetherin in cell-to-cell transmission *in vivo*.

**Figure 3:**
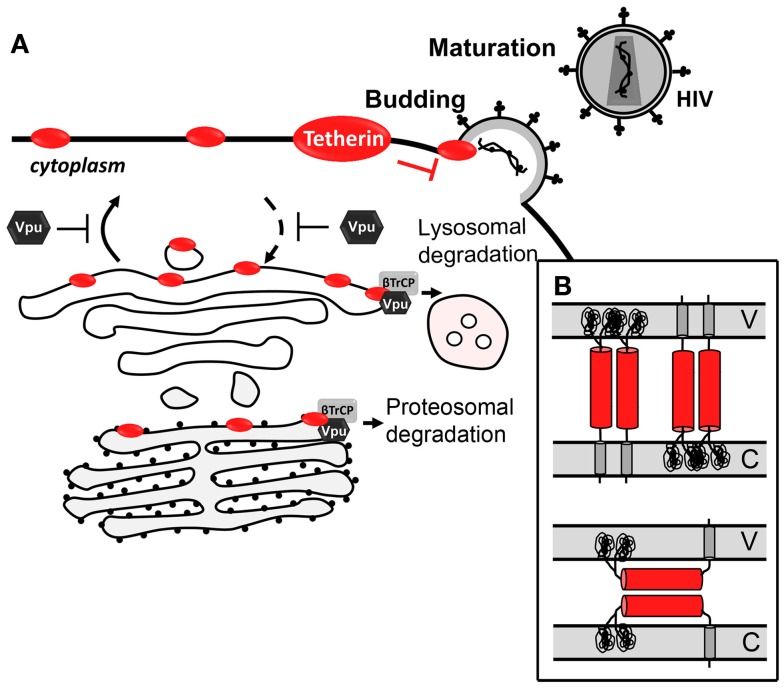
**Antiviral mechanism of tetherin**. **(A)** Tetherin prevents Vpu-defective virus release by tethering newly formed virus to the cell-surface. Vpu counteracts tetherin by trapping it at the TGN or targeting it for proteasomal or lysosomal degradation. **(B)** Topological models for antiviral tetherin mechanisms. One end of tetherin is anchored at the cell plasma membrane and the other end is anchored at the surface of the virion (top); or both ends of one monomer of tetherin parallel homodimers are inserted in either the cell plasma membrane or the viral membrane (bottom). V stands for viral membrane and C stands for cellular membrane.

Several observations strongly support that tetherin prevents virion release through a direct mechanism, which involves a physical binding between the host cell and the virion (Figure 3A). First, tetherin was shown to localize between the cell and the virion (239, 259–261). Secondly, its antiviral activity could be mimicked by a synthetic protein, with low amino acid sequence similarity but similar topology containing two-membrane anchors at either end and a coiled-coil ectodomain in between (261). Two topological models for tethering activity are suggested (Figure 3B) [reviewed in Kuhl et al. (262)]. Viruses escape tetherin restriction in a species-specific manner. Vpu is the HIV-1 viral antagonist of tetherin (251, 252). This viral protein is a small transmembrane protein that interacts with tetherin, through its respective transmembrane domains, trapping it at the TGN or targeting it for degradation through proteasomal or lysosomal pathways (263–268). Both situations lead to a decrease of tetherin levels at the plasma cell membrane and thus at the HIV-1 assembly sites. HIV-2 lacks Vpu, but it counteracts tetherin antiviral activity through its Env protein in a similar way. HIV-2 Env diminishes tetherin levels at the plasma membrane by sequestering it at the TGN, while the total cellular levels of tetherin remain unaltered (269, 270). Besides HIV-1 Vpu and HIV-2 Env, SIV Env, SIV Nef, SIV Vpu, KSHV K5, and Ebola envelope glycoprotein have also been shown to thwart tetherin through distinct mechanisms (253).

## Tetherin Clinical Studies and Tetherin-Based Therapeutics

With few clinical studies on tetherin, its impact in disease progression and in LTNPs is still largely unknown. So far, no significant differences in tetherin expression levels were found between HIV-1-exposed seronegative subjects and healthy controls (271). However, tetherin expression was shown to be increased in mononuclear leukocytes, including CD4^+^ T lymphocytes, from untreated HIV-positive patients when compared to cells of uninfected controls during the acute phase of infection (271, 272). This increase was more pronounced during the acute phase of infection (272). In addition, it was also reported that subjects under anti-retroviral treatment, who present reduced viremia, also exhibit total tetherin protein levels similar to those observed in uninfected controls (271, 272). Altogether, these observations suggest that tetherin, as an interferon-stimulated gene, can be involved in the control of the acute phase of infection. However, as the disease progresses it becomes inefficient. A role of tetherin in IFN-α pathway in HIV-1 infection is further supported by the observation that pegylated IFN-α/ribavinin combination therapy for HIV/hepatitis C virus co-infected individuals decreased HIV-1 viral load, which is correlated with an increase in tetherin levels in CD4^+^ T cells (273). The notion that tetherin is a part of the IFN pathway to control HIV infection *in vivo* was further investigated *in vitro*. PBMCs treated with IFN-α show an increase in the expression of tetherin to levels high enough to counteract the Vpu protein, resulting in the viral tethering that blocks the release of wild type HIV virions (272). *In vitro* and *in vivo* results led to the proposal that induction of tetherin expression may be a valid therapeutic approach in the fight against AIDS (272, 273). The easiest way to induce tetherin expression would be to treat patients with IFN-α. However, not only is IFN-α treatment accompanied by several side-effects, it was also associated with an increase in AIDS progression (274). A more effective and safer therapeutic approach should not increase tetherin expression levels, but, rather, increase its functional levels at the cell-surface by competitively inhibiting its interaction with Vpu. This could be achieved by peptides or other type of molecules with higher affinity for the transmembrane domain of Vpu than the transmembrane domain of tetherin. This therapeutic strategy has now been supported by a recent *in vitro* study, where the expression of the tetherin delGPI mutant inhibited the release of HIV-1 wild type from tetherin-positive HeLa cells by competitively inhibiting the interaction of Vpu with endogenous tetherin through its transmembrane domain (275). *In vitro* and *in vivo* studies with other inhibitors should now follow to fully understand if inhibition of Vpu/tetherin interaction is a viable therapeutic approach to control HIV spread in infected individuals.

## SAMHD1

The sterile alpha motif (SAM) and histidine-aspartate (HD) domain-containing protein 1 (SAMHD1) contains a SAM and a HD domain in tandem. SAM domain-containing proteins putatively interact with other proteins and RNA (276, 277). The SAM domain of SAMHD1 protein harbors a nuclear localization signal (^11^KRPR^14^) within the first 15 amino acids of the protein sequence that specifically localizes SAMHD1 to the nucleus (278–280). However, SAMHD1 also localizes in the cytoplasm of resting and activated CD4 T cells and macrophages (281, 282). The HD domain is found in a superfamily of proteins with a metal-dependent phosphohydrolase activity (283). Enzymatic and structural studies showed that this domain is the sole determinant of the activity, oligomerization and RNA binding activity of SAMHD1 (280, 284). SAMHD1 is expressed in a variety of tissues at different levels. It is highly expressed in myeloid-derived cells, such as monocytes, macrophages, DC, and resting CD4 T cells (naïve, central memory, and effector memory). These cells are highly refractory to HIV-1 infection, supporting the role of SAMHD1 as a RF. SAMHD1 expression is independent of the cell activation state (281), is inducible by type-I IFN in monocytes (285) and is transiently sensitive to type-I IFN in DC (286). The transient response of DC to type-I IFN consists of an increase in SAMDH1 mRNA levels early after IFN treatment that do not result in protein expression (286). SAMHD1 appears to be part of an immune evasion strategy to escape antiviral responses mediated by the detection of dsDNA by dsDNA-sensors (dsDNA-sensor antiviral responses). SAMHD1 is a deoxynucleotide triphosphate (dNTP) hydrolase that is activated by the binding of GTP to its allosteric site, cleaving dNTPs into deoxynucleoside and triphosphate products (284, 287). Consequently, SAMHD1 reduces the intracellular dNTP pool below levels that support HIV-1 reverse transcription, blocking HIV-1 replication and avoiding the induction of IFN responses (281, 288–290). This phenotype can be reverted by addition of deoxynucleotides to the culture medium or by transduction of cells with the HIV-2 accessory protein-Viral Protein X (Vpx). In cellular models using primary SIV, SAMHD1 restriction can also be overcome by transduction of cells with the Viral Protein R (Vpr) (289, 290).

**Figure 4:**
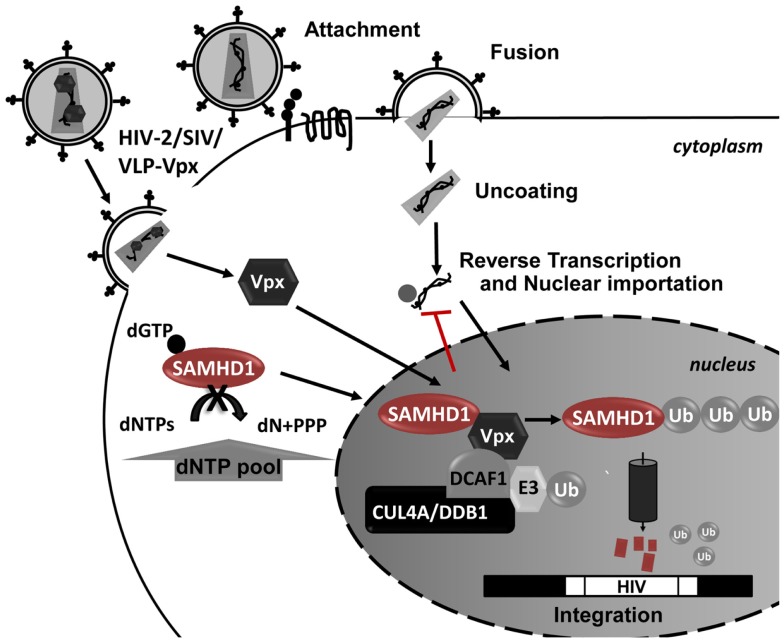
**Schematic representation of SAMHD1 Vpx-mediated proteosomal degradation during HIV-1 infection**. In the presence of Vpx, SAMHD1 is recruited to a CUL4-DDB1-DCAF1 protein complex in the nucleus, leading to the proteosomal degradation of SAMHD1. The SAMHD1 reduction leads to an increase in the dNTP pool allowing the HIV replication in these cells.

Vpx is a small lentiviral accessory protein with 12–16 kDa that is packaged into the budding virion during assembly, allowing it to act on newly infected cells prior to proviral integration. In the host cell, Vpx is translocated into the nucleus, where it associates with the cullin-4A-ring-E3-ubiquitin ligase (CRL4) (Figure 4). The formation of this complex leads to degradation of SAMHD1 restriction factor by the 26S proteasome (281). While SAMHD1 activity as a restriction factor is independent of its subcellular location, Vpx is ineffective against cytoplasmic SAMHD1 (279). SAMHD1 proteosomal degradation causes an indirect increase in the intracellular pool of dNTPs, allowing HIV-1 reverse transcription and the infection of cells that are otherwise very resistant to the virus. For example, full-length viral cDNA accumulates in resting CD4 T cells and HIV-1 infectivity is restored in monocyte-derived macrophages (MDMs) and DC cells upon Vpx expression and SAMHD1 degradation (279, 281, 286, 288–292). However, these cells possess an unknown sensor in their cytoplasm that detects newly synthesized viral proteins and triggers IFN responses (293). Therefore it is conceivable that HIV-1 did not evolve an anti-SAMHD1 counteracting protein to avoid the cellular detection of viral proteins and consequent immune activation.

SAMHD1 activity is not correlated with its expression levels, as only resting CD4 T cells restrict HIV-1 infection (288). This observation and the identification of naturally occurring splice variants of SAMHD1 suggest a post-transcriptional regulation of SAMHD1 activity (288, 294). SAMHD1 mutations are associated with rare genetic disorders including the Aicardi-Goutieres syndrome (AGS). AGS reproduces a biologic state of viral infection due to excessive production of IFN-α and increased immune activation (295). In addition, monocytes from AGS patients are highly susceptible to HIV-1 infection (285), and mutations that block hydrolase activity result in the loss of SAMHD1 antiviral activity (278). Viral RNA binding to SAMHD1 increases its dNTP hydrolase activity, leading to a rapid elimination of DNA intermediates and the subsequent blockade of HIV-1 replication. Thus, SAMHD1 seems to play a key role in a strategy of the immune system to avoid immune cellular responses upon viral infection.

## SAMHD1 Clinical Studies and SAMHD1-Based Therapeutics

There is evidence that naturally occurring splice variants destabilize SAMHD1 leading to its rapid elimination (294). Such evidence highlights the role of alternative regulatory pathways to control of HIV-1 infection, such as miRNA or post-transcriptional modifications; and the potential relationship between alterations in SAMHD1 activity and disease progression. The therapeutic manipulation of intracellular dNTP pools and the development of SAMHD1 inhibitors to trigger the innate immune response have been suggested as potential anti-retroviral therapies (281, 282). However, there is a lack of clinical studies in this direction, and consequently a direct association between SAMHD1 and HIV-1 infection remains to be established. A recent study evaluated the association between SNPs and SAMHD1 expression and activity (296). In this study the authors identified a SAMHD1 SNP, where an A/G substitution occurred at position 59885 (rs1291142), which significantly interfered with the expression of SAMHD1 in B cells from 70 healthy donors. However, they found that this SNP is not present in the genome-wide study of HIV-1 controllers and non-progressors from the larger published European and African-American cohorts (296). The authors consequently claim that this SAMHD1 polymorphism probably does not contribute to the control of HIV-1 infection. However, this conclusion is based on their observations of a very small cohort of healthy subjects. LTNPs and HIV controllers are very rare in the general population since most people develop AIDS upon HIV-1 infection. Therefore, in such a small sample it is very unlikely to have a LTNP/controller and, by extension, to identify a SNP in SAMHD1 that could be associated to this phenotype. In summary, the connection between SAMHD1 variants and HIV-1 progression should be further tested in clinical studies. We believe it could provide insight into novel therapeutic strategies against HIV.

## Conclusion/Final Remarks

The existence of rare individuals who resist infection, delay the disease outcome or control viral replication without the need of anti-retroviral therapy demonstrates that prevention of infection and long-lasting disease remission are attainable objectives. A large group of factors contributes to the balance between viral replication and host antiviral response, including cell-specific RF and “defects” in naturally occurring helper factors.

Despite latest advances in the HIV-1 field, our knowledge on how LTNPs and ECs control HIV-1 infection is still limited. The few genes whose alterations have been found to be correlated with different rates of disease progression cannot fully account for the differences observed among the patients. Thus, the host RF and genetics responsible for AIDS delay in non-progressors remain to be identified. Here, we reviewed a series of polymorphisms and expression alterations in HIV-helper factors that are related to control of HIV-1 infection, with special focus on those RFs that counteract HIV-1 entrance into and exit from the host cell. Genome-wide genetic association studies confirmed some of the genetic-variants such as CCR5Δ32 and HLA-B5707 (36) but were not able to pinpoint a single gene common to all LTNPs and responsible for slow disease progression. The choice of very restrictive statistical thresholds or gene chip arrays that do not cover all gene variants or non-coding DNA sequences may exclude relevant host factors from the analysis. However, AIDS is a complex disease and the different rates of disease progression observed may be also due to a combination of multiple factors.

So far, among all identified genetic factors playing a major role in the HIV decrease, the CCR5Δ32 polymorphism remains the main genetic factor conferring human resistance to HIV-1. Display of chemokine receptors at the cell-surface and their conserved structure make them amenable targets for drug development. This is the case for maraviroc and novel gene therapy approaches that are currently used for the treatment of AIDS or are currently being tested in ongoing clinical trials. This example of success in translational research highlights the relevance of a better understanding of how LTNPs control disease progression for the development of new therapeutic tools to cure AIDS. We expect that, in the near future, other drugs or gene-based therapy approaches targeting other host proteins (helper or RFs) will enter clinical trials and translate to the clinic.

## Conflict of Interest Statement

The authors declare that the research was conducted in the absence of any commercial or financial relationships that could be construed as a potential conflict of interest.
